# Association between Urban Greenspace and Health: A Systematic Review of Literature

**DOI:** 10.3390/ijerph18105137

**Published:** 2021-05-12

**Authors:** Vincenza Gianfredi, Maddalena Buffoli, Andrea Rebecchi, Roberto Croci, Aurea Oradini-Alacreu, Giuseppe Stirparo, Alessio Marino, Anna Odone, Stefano Capolongo, Carlo Signorelli

**Affiliations:** 1 School of Medicine, University Vita-Salute San Raffaele, 20132 Milan, Italy; gianfredi.vincenza@hsr.it (V.G.); croci.roberto@hsr.it (R.C.); oradini.aurea@hsr.it (A.O.-A.); stirparo.giuseppe@hsr.it (G.S.); marino.alessio@hsr.it (A.M.); c.signorelli@hsr.it (C.S.); 2 Architecture, Built Environment and Construction Engineering Department, Politecnico di Milano, 20133 Milan, Italy; maddalena.buffoli@polimi.it (M.B.); stefano.capolongo@polimi.it (S.C.); 3 Department of Public Health, Experimental and Forensic Medicine, University of Pavia, 20158 Milan, Italy; anna.odone@unipv.it

**Keywords:** physical activity, mental health, depression, anxiety, stress, green areas, green infrastructures, urban greenery, urban health, non-communicable diseases

## Abstract

The current review aimed to explore the association between urban greenspaces and health indicators. In particular, our aims were to analyze the association between publicly accessible urban greenspaces exposure and two selected health outcomes (objectively measured physical activity (PA) and mental health outcomes (MH)). Two electronic databases—PubMed/Medline and Excerpta Medica dataBASE (EMBASE)—were searched from 1 January 2000 to 30 September 2020. Only articles in English were considered. Out of 356 retrieved articles, a total of 34 papers were included in our review. Of those, 15 assessed the association between urban greenspace and PA and 19 dealt with MH. Almost all the included studies found a positive association between urban greenspace and both PA and MH, while a few demonstrated a non-effect or a negative effect on MH outcomes. However, only guaranteeing access is not enough. Indeed, important elements are maintenance, renovation, closeness to residential areas, planning of interactive activities, and perceived security aspects. Overall, despite some methodological limitations of the included studies, the results have shown almost univocally that urban greenspaces harbour potentially beneficial effects on physical and mental health and well-being.

## 1. Introduction

Nowadays, humans live in a predominantly urban world. Between 1990 and 2000, the number of people living in urban areas rose by 25% [1]. Worldwide forecasts estimate that 6 out of 10 people will live in cities by 2030, a figure that will reach 8 out of 10 by 2050 [2]. This progressive increase has led the scientific community to explore and assess the urban environment’s salutogenic effects [3]. On the one hand, urbanization has improved populations’ health status, thanks to better career and education opportunities, and increased access to essential healthcare services [4,5]. On the other hand, rapidly growing cities pose new public health threats. Among those is the increase in social inequalities and lifestyle-related risk factors, such as lack of physical activity and unbalanced dietary habits [6,7], pollution and traffic, and the environmental degradation of natural areas [8]; which, in turn, increase the incidence of a vast spectrum of diseases and conditions [9,10]. Overcrowding exacerbates the risks of communicable diseases (CD), as shown by the COVID-19 pandemic [11,12,13]. Urbanicity might also represent a risk factor for chronic non-communicable diseases (NCD) and other leading causes of death and disability, such as, for instance, road traffic injuries and violent crimes. As cities exploit a large share of the world’s natural resources, they account for a considerable contribution to climate change-related health issues [14,15]. Urbanization’s overall health impact also depends on specific populations’ elements of vulnerability and resilience, their ability to adapt to environmental changes, on health services organization and urban planning. In this perspective, the idea that urban green areas might exert health benefits dates back to the early 1800s. Healthcare organizations such as the Commons Prevention Society and the National Health Society started advocating for the creation of publicly accessible urban green spaces, describing them as “the lungs of the city” [16].

In more recent times, the World Health Organization (WHO) Regional Office for Europe has launched a “WHO European Healthy Cities Network”, which embodies a “Healthy Cities” vision. Moreover, referring to the “Urban Health Rome Declaration” at European meeting “G7 Health”, which defines the strategic aspects and actions to improve Public Health into the cities, and referring to the Agenda 2030, in which the 11th Sustainable Development Goal (SDG) argues about “Sustainable Cities and Communities. Make cities and human settlements inclusive, safe, resilient and sustainable”, one of the most expressive syntheses of the challenging relationship between urban planning and Public Health is stated by World Health Organization (WHO, 2016): “Health is the precondition of urban sustainable development and the first priority for urban planners”. According to the project’s programmatic framework, “cities’ healthiness level is indicated “by a process, not an outcome”. The Network defines “a healthy city” as “one that continually creates and improves its physical and social environments and expands the community resources that enable people to mutually support each other in performing all the functions of life and developing to their maximum potential” [17]. Several studies have shown that green areas can improve general well-being [18], self-perceived health status [19,20], increase physical activity (PA) levels [21,22], curb morbidity and rise life expectancy [23], satisfaction with their housing situation, jobs, and life perspectives [24]. However, the evidence is still somehow ambiguous. Previous research failed to univocally and conclusively demonstrate the beneficial effect of urban green space on both physical and mental health [25,26]. This is probably due to high heterogeneity in the population’s characteristics, study period, sample size and study design, but also due to the green area and infrastructure features included and analyzed.

In light of the above considerations, the current review’s broader objective was to explore the association between urban greenspaces and health indicators. The specific aim was to analyze the direction and strength of the association between urban greenspaces exposure and two selected health outcomes: objectively measured PA, and mental health (MH) outcomes in Organization for Economic Co-operation and Development (OECD) countries. Our ultimate goal was to critically appraise the available evidence so as to offer material to inform future community-based urban planning strategies and public health policy initiatives.

## 2. Materials and Methods

The methods for this systematic review were designed following the Cochrane Collaboration’s recommended approach [27]. We conducted each phase of the study and reported its results according to the Preferred Reporting Items for Systematic Reviews and Meta-Analysis (PRISMA) [28] and the Meta-analysis Of Observational Studies in Epidemiology (MOOSE) [29] guidelines.

### 2.1. Search Methods for Study Retrieval

Studies were retrieved by searching two electronic databases, PubMed/Medline and Excerpta Medica dataBASE (EMBASE). The search strategy was developed in September 2020 by pooling predetermined keywords launched at first on PubMed/Medline and then adapted for EMBASE. Whenever possible, controlled vocabulary thesauruses—PubMed’s MeSH (Medical Subject Headings) and EMBASE’s Emtree—were used to explore broader content. Items were logically combined with the Boolean operators “AND”, “OR” and “NOT”. The full search strategy is available in Supplementary Table S1. The list of references was also screened to identify any additional eligible studies. Finally, experts in the field were consulted. We developed a standardized protocol to identify the research question, formulate the search strategy, set inclusion and exclusion criteria and select quality appraisal tools for primary studies. The protocol was shared and discussed within the research team and fully approved before starting the review.

### 2.2. Inclusion and Exclusion Criteria

Since we focused on the association between urban greenspaces objectively measured physical activity (PA) and mental health (MH), we only included original papers measuring PA objectively through accelerometer, pedometer, video recording or similar devices. For MH outcomes, we assessed a plurality of domains, including, but not limited to, the most prevalent MH disorders, such as depression, anxiety, and psychosocial stress. Outcomes could be calculated as continuous or dichotomic, indifferently. Moreover, we accepted both self-reported measures and data extracted from clinical databases and repositories or self-assessed by interviews for MH outcomes. As for publicly accessible of urban greenspace exposure, we referred to the general definition reported in 2016 by the WHO Regional Office for Europe (EURO): “public green areas used predominantly for recreation such as gardens, zoos, parks and suburban natural areas and forests, or green areas bordered by urban areas that are managed or used for recreational purposes” [30]. However, we also relied on a more detailed definition issued by a 2017 EURO brief for action [31]. We finally synthesized the theoretical framework with extensive consultation of experts in the field. Details are provided in Supplementary Table S2.

Furthermore, to improve the internal validity, we set a geographic limit, including only studies conducted in the OECD area. We also opted for a language limit, selecting only articles published in English. Lastly, we adopted a time limit, filtering for studies after 2000. We used this time limit for several scientific reasons. Firstly, the availability of techniques to objectively measure PA dates back to the last 10 to 15 years. Therefore, we judged it implausible to find older studies meeting our pre-fixed criteria. A recent systematic review indirectly confirms our hypothesis, since the earliest study assessing the association between objectively measured PA and depression was published in 2004 [32]. Secondly, OECD’s urban areas have known profound changes over the last 20 years. Besides, the psychiatric nosography itself has evolved, with updates to many diagnostic criteria. Therefore, we assumed that extending the time frame of our research indiscriminately could undermine its results, with the concrete risk of collecting heterogeneous, poorly comparable data for both outcomes.

Finally, we excluded all non-original studies (e.g., reviews, book chapters, correspondence, brief notes, commentaries, conference proceedings, abstracts). Supplementary Table S3 shows a detailed description of inclusion and exclusion criteria for both observational and interventional studies, developed in accordance with the Population, Intervention/Exposure, Comparison, Outcomes and Study design (PEOS), adjusted for observational studies, and extended with time and language filters, as recommended by the Cochrane Collaboration [33].

### 2.3. Study Selection, Data Extraction and Quality Evaluation

All identified records were analyzed in a two-step process. First, three researchers (G.S., R.C., A.O.-A.) independently screened titles and abstracts to assess potential eligibility; then, eligible studies were evaluated in full. A pre-defined, customized spreadsheet was used to extract and collect useful data (Microsoft Excel^®^ for Windows Redmond, WA, USA, 2007). As carried out before [34], to reduce methodological heterogeneity and to standardize data extraction, the spreadsheet was pre-piloted by four researchers (V.G., G.S., R.C., A.O.-A.) on 10 randomly selected records. Disagreements were solved by discussion among the three researchers involved in the study selection (G.S., R.C., A.O.-A.), or by the decision of a fourth (senior) researcher (V.G.).

As carried out in previous systematic reviews [35,36,37], both qualitative and quantitative data were extracted from the original studies. Qualitative data recorded included the following items: name of the first author, year of publication, study period, country, study design, type of urban greenspace analyzed, city where the study was conducted, statistical analysis performed, tool used to measure PA or MH, and outcomes domain (for PA, we differentiated between PA generally performed or performed in the greenspace analyzed; for mental health, we specified which type of condition was assessed, e.g., depression, anxiety, stress, etc.). Moreover, when available, sociodemographic characteristics of the subjects were recorded (e.g., age, gender). The quantitative data extracted included: sample size, and the most relevant results quantifying the association between urban greenspace and PA or MH. For studies displaying incomplete or partial data, the corresponding author was reached via e-mail for clarifications.

The quality evaluation of the included publications was carried out independently by three authors (A.M., G.S., and A.O.-A.) using the New-Ottawa Scale (NOS) for observational studies [38] and the Risk of Bias-2 (RoB-2) of the Cochrane Collaboration tool for randomized trials [39]; the National Institute of Health quality assessment tool for pre-post intervention studies [40], as suggested by Ma et al. [41]. However, since the NOS did not provide a checklist for cross-sectional studies, we used a modified version [42], adapted to perform a quality assessment of cross-sectional studies. We also used the NOS to assess the methodological quality of quasi-experimental studies, due to their observational nature. We used the 15-item checklist proposed by Dufault and colleagues for ecological studies [43]. Referring to the NOS, the maximum quality score (QS) is 9, categorized as follow: QS > 7 high quality, 5 < QS ≤ 7 moderate quality, and QS ≤ 5 low quality. For the quality assessment of randomized trials, the evaluation only allows for a quality judgment without quantitative results ranging between high risk of bias, some concern and low risk of bias. This is the same also for pre-post intervention, for which the judgment can be good (if score ≥ 75%), fair (score between 75% and 25%), and poor (if score ≤ 25%). Regarding the QS suggested by Dufault et al. for ecological studies, the maximum score is 21 points, of which QS ≤ 7 was considered low quality, 7 < QS ≤ 14 was considered moderate quality and lastly QS >14 was considered high quality.

## 3. Results

### 3.1. Literature Search

**Figure 1:**
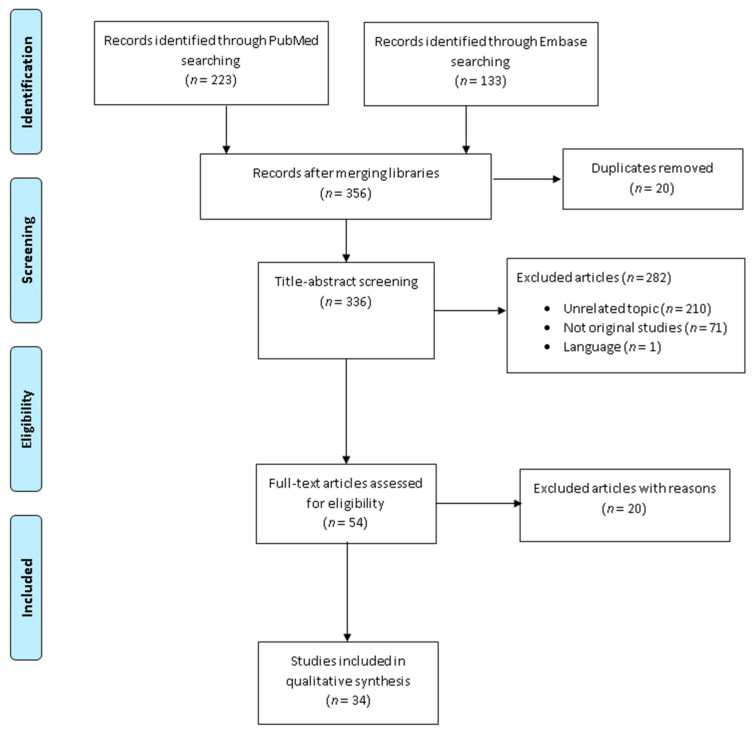
Flow diagram of the selection process.

A total of 356 records were initially retrieved by the literature search. After duplicate removal, 336 records were left for the title-abstract screening. Based on the title and abstract, 282 articles were removed, while the remaining 54 were screened by reading the full-text. In the second screening step, 20 articles were eliminated, and the reasons for removal listed (Supplementary Table S4) [44,45,46,47,48,49,50,51,52,53,54,55,56,57,58,59,60,61,62,63]. Finally, 34 articles met all the inclusion criteria and were thus incorporated into the qualitative synthesis [64,65,66,67,68,69,70,71,72,73,74,75,76,77,78,79,80,81,82,83,84,85,86,87,88,89,90,91,92,93,94,95,96,97]. Figure 1 shows the selection process. The quality evaluation of the included studies is reported in Supplementary Table S5. Most of the observational studies were judged as high quality. In contrast, the interventional studies show some concerns for risk of bias.

### 3.2. Characteristics of Included Studies

Overall, the articles’ study period spanned 19 years, from 2000 [79] to 2019 [85]. Almost all the included studies (31/34, 91%) were based in a single country. Half of those (19/34, 55%) were set in English-speaking countries (12 United States of America [71,72,73,74,77,82,83,88,92,93,94,95], four United Kingdom [48,56,58,64], one Canada [75], one Australia [66], one New Zealand [81]). European and Asian countries were involved in 29% (10/34) of the articles (three Lithuania [45,47,67], two Netherlands [96,97], one Denmark [64], one Norway [79], two Japan [85,91], and one South Korea [80]). South America was the least represented continent, with only two studies, which both took place in Colombia [69,86] (Table 1). The remaining three studies were multi-country based. One [70] investigated the association between circadian variation patterns of moderate-vigorous PA and total parks number in 10 countries. A second article explored the relationship between PA’s quantity and urban environment features in fourteen OECD countries’ cities [90]. Finally, a third study considered mental health indicators measured by the MHI-5 (Mental Health Inventory-5) scale and urban greenspace characteristics in four European cities [89]. As for the study design, 26 were observational; of them, almost all (23/34, 67%) were cross-sectional [65,67,68,69,70,74,75,77,78,79,80,83,84,86,87,88,89,90,93,94,96,97]; the remaining were one cohort [66] and two ecological study [81,82]. The other eight studies were experimental, with differences in nature. Five of them were pre-post intervention [44,56,65,71,75], two were randomized [73,92] and the last one was quasi-experimental with only assessment post-intervention [72]. For this reason, the latter was assessed as a cross-sectional study (as reported in Supplementary Table S5). Approximately half of the included studies (14/34, 41%) assessed the health effect of parks and urban meadows (PUM) selectively [45,47,50,51,52,53,54,55,56,62,64,65,67,72]; the other eleven studies combined PUM with other types of urban green areas [64,68,69,77,79,80,81,88,93,94,97] (details in Table 1); three studies assessed the association between recreational and urban gardening facilities (RUGF) and PA [63,66,70]; three studies assessed the impact of small urban greenspaces (SUG) on health outcomes [71,75,76]; one study evaluated the health-related effect of neighbourhood green spaces (NGS) [89], one article assessed the total urban greenspace [66]. One single article did not specify the type of urban greenspace [78].

### 3.3. Tools Used to Assess Health Outcomes

**Figure 2:**
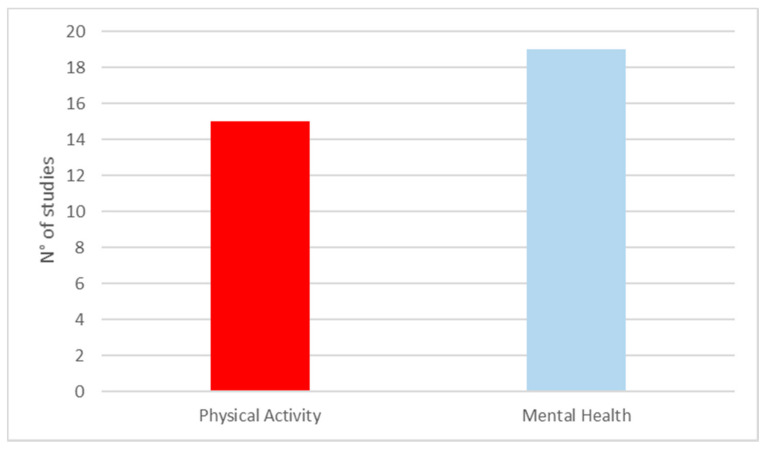
Number of articles stratified by health outcome (Physical Activity (PA) or Mental Health (MH)).

PA outcomes were analysed by 15 articles [64,70,71,72,73,74,75,82,83,86,88,90,93,94,95] (Figure 2). The majority of those studies (11/15, 61%), dealt specifically with urban greenspace-based PA [71,72,73,74,75,82,83,86,93,94,95]. In contrast, a third of them (4/15, 33%) reported overall data about the total amount of PA practised, regardless of the setting [44,50,68,70]. To objectively measure PA, the majority of the studies used some kind of video recording system. In more detail, nine used the System for Observing Play and Recreation in Communities (SOPARC) [51,52,53,54,55,63,66,68,75], two used the System for Observing Play and Leisure Activity in Young (SOPLAY) [93,94], whereas four studies used the accelerometer, alone [50,62,70] or in combination with GPS and GIS [64].

MH outcomes were considered by 19 records [65,66,67,68,69,76,77,78,79,80,81,84,85,87,89,91,92,96,97]—all of which seem to adopt a unified analytic approach. Indeed, they evaluate multiple MH domains in parallel. Public MH research has clearly demonstrated high prevalence rates of comorbidity in people living with MH disorders [98]. In community surveys of the general population, findings of several areas of psychologic dysfunction or self-perceived discomfort are common [99]. Well-being and quality of life were the most frequently assessed MH outcomes (5/19, 26%) [45,47,49,56,58], followed by depression (3/19, 16%) [60,67,69], stress (4/19, 23.5%) [46,57,60,64], general mental health (4/19, 23.5%) [59,72,76,77], anxiety and mood state (3/19, 16%) [61,65,71], and suicide [68]. The total number of MH outcomes assessed is higher than the total included studies, because most of them assessed more than one outcome at once. All the MH dimensions were assessed by specific psychometric scales, often validated by the latest edition of the Diagnostic and Statistical Manual of Mental Disorders (DSM-V) [100]. Two studies analysed MH outcomes by an unvalidated questionnaire [79,80], a record linkage [81], and another one with purely epidemiologic methods [68]. In the latter, authors used a Poisson linear regression model to describe the relationship between cause-specific mortality rates for suicide in the general population and 50 English cities’ greenspace coverage [68].

### 3.4. Greenspace and Physical Activity

Across all the included studies, a positive association was found between urban greenspaces exposure and PA levels. Main predictors of enhanced PA were: presence of urban greenspaces in a 0.5 to 1 km radius from the subjects’ homes [90], total number of urban greenspace in the neighborhood, and their accessibility through public transport [70]. In a study analyzing circadian variations in PA patterns, PA levels peaked in the afternoon (2 to 5 p.m.) and where much lower in the evening and night [70]. Urban greenspaces with playgrounds are effective enablers of increased PA intensity in children [93]. However, this urban greenspace feature displayed poorer results in more deprived city neighborhoods [93,94]. Globally, rural [88] and low-income neighborhoods had diminished use rates [82]—even more when disaggregating data by sex, with women being the less frequent users [72]. Interestingly, the same 2014 study highlighting different rates of women users also found an inverse relationship between park size, visitors and PA intensity. On average, pocket parks had higher visitors, but less reported PA intensity than broader-sized urban greenspaces [72].

One study concluded that exercise facilities and related amenities in urban greenspaces promote PA across demographics, especially in women [86]. Besides providing public access to [83] and ensuring regular maintenance [95] of urban greenspaces, the total number and variety of working equipment [75], and scheduled plans for sports activities are other aspects that need to be factored in [71].

A randomized study with four arms as follows: arm (1) free PA classes; arm (2) a prize contest based on the number of park visits; arm (3) interventions of arms 1 and 2, combined; arm (4) no intervention; showed that the most significant increase in PA was reached in arms 1 and 2 [73]. Walking loops proved effective in boosting PA levels and incrementing the total number of urban greenspace visitors [74]. Two studies investigated the effects of urban greenspace renewals on citizenship perception, engagement and use. The first article’s setting were low-income neighborhoods in San Francisco (USA) [95]. The scholars proved that, after renovations were carried out in two urban greenspaces, the average number of adult users increased between four and nine times. A 2017 Danish study presented a project of integrated urban rebuilding. Four new UGSs were created in a low-income area in Copenhagen [64]. The authors report an increase in the average daily time spent by adolescents in practising PA (+4.5 min/day, *p* < 0.05) [64].

### 3.5. Greenspace and Mental Health

Only three out of the 19 included MH-related articles did not find a statistically significant association between the urban greenspace and mental health. A study comparing greenspace coverage to the cause-specific mortality rates for suicide in England (between 2002 and 2009) reported no association between increasing quintiles of greenspace coverage and age-standardized mortality risk ratios for suicide [68]. Similarly, no statistically significant association was found between urban greenspace use in all (four) European cities, except for Barcelona, where living in ‘greener’ spaces was associated with higher Mental Health Inventory-5 (MHI-5) scale scores [89]. Lastly, Ihlebaek et al. did not find a statistical association between MH disorders and urban greenspace exposure in men, but only in women in a border-line inverse association [79].

All the remaining included studies found a positive association between urban greenspace exposure and MH. Specifically, four studies considered psychosocial stress, alone [77] or in combination with other mental health outcomes [80], both in adolescents and adults. The main predictors of lower-level stress were a higher number of urban greenspaces and easier accessibility, higher tree density, and the possibility of performing leisure activities (both physical and intellectual). In particular, higher number and easier accessibility were associated with lower levels of stress in both adolescents (in Buffalo and New York) [77], and elderly (over 65 years old) [80]. The latter also benefited from a lower level of depression [80]. A cohort study showed that higher tree density in the neighbourhood was associated with a lesser degree of psychological distress among adults (Australia) [66]. Lastly, two studies carried out a separate analysis of different activities performed in urban greenspace to disentangle their relative contributions to mental well-being and distress [76,84]. In a first article, people going to urban greenspace to perform leisurely activities experienced significantly lower psychological distress than their non- urban greenspace dweller counterparts [84]. In a second study by Coventry and colleagues, various intellectual and motor activities proved effective in reducing stress levels in the exposed subgroup [76].

One study was specifically focused on depressive symptoms [87], while the other assessed both general mental health and depression. The first one was a Lithuanian study that indicated an inverse relationship between individual-level depressive symptoms and residential distance from urban greenspaces, which was more marked in women [87]. The second, was a USA article exploring the effect of a social gardening program performed in vacant urban greenspaces located in neighbourhoods with average income levels below the poverty threshold. There were significantly lower depressive symptoms after exposure [92], but failed to demonstrate a significant improvement of the general mental health. On the contrary, the other two studies assessing the impact of urban greenspace on general mental health found a positive association between higher number and easier accessibility of urban greenspace among adults, in the Netherlands [96,97].

Four studies dealt with mental well-being/quality of life in adults and children. The two analysing the paediatric population showed how lower urban greenspace attendance rates were associated with increased risk of MH issues [65], where lower maternal education level represents an additional risk factor [67]. A third study based in England was conducted in a sample of adults. The authors showed that a lack of urban greenspace access was significantly associated with worse mental well-being [78]. One study conducted in Colombia considered the effect of urban greenspace on quality of life metrics [69]. Urban greenspace accessibility, maintenance status, and perceived security were associated with higher quality of life metrics and lower anxiety and depression levels.

Three studies explored urban greenspace’ effect on anxiety. Song and co-authors [91] measured anxiety-related symptoms in two groups of citizens after 15 min of walking in urban greenspaces, as opposed to urban built environments. In the second study, anxiety levels dropped after the subjects were exposed to natural landscapes [85]. In an ecological study, anxiety decreased for reduced urban greenspace distance [81].

## 4. Discussion

The current systematic review has identified a total of 34 studies. Of those, 15 investigated the effect of urban greenspace exposure on PA and 19 on MH. Specifically, only a small fraction of these demonstrated a non-effect or a negative impact on MH outcomes. On the contrary, the majority reported a beneficial effect on different MH aspects, such as levels of self-perceived stress, depressive symptoms and perceived mental well-being. The same results were reached for PA. All the studies showed that exposure to urban greenspaces increased PA. However, what emerged is that both health outcomes improved substantially with the exposure to well-kept urban greenspaces. Maintenance has also proven to be a therapeutic activity for people with MH issues. In this perspective, the study by South et al. [92] highlighted how users’ involvement in abandoned urban greenspaces’ renewal and maintenance, particularly in economically deprived settings, can act as a surrogate mood-stabilizing therapy for people with depressive disorders. Many recent pieces of evidence are coherent with our results, identifying green space as an important factor impacting on both physical and mental health [101,102,103]. In particular, Wendelboe-Nelson et al. stressed the importance of incorporating green space during city planning and in public health policies, especially considering the world’s growing urban population [101].

Emotional well-being is an essential aspect of overall health. Among young people, emotional well-being helps develop intrapersonal and interpersonal relationships, with a long-term influence on health trajectories, both in adulthood and later life stages [104]. Its absence causes physical and MH problems. Due to the growing burden of mental disorders in children and adults, the WHO has called on increasing knowledge levels of emotional well-being determinants [105,106,107]. The complex and articulated relationship linking urban greenspaces, emotional well-being, and health benefits involve individual characteristics and social and physical environments’ features [108,109]. Actually, even the paucity of the literature, Wendelboe-Nelson et al. in their work found that green spaces may affect health in different ways and with different benefits based on population’s characteristics (e.g., socio-economic status, age, and sex) [101]. However, as confirmed by Lee et al., evidence is limited, especially in understanding the amount of urban green space exposure and the related beneficial effects [102]. Moreover, heterogeneous results have been found on how users’ characteristics might impact on urban green space usability and consequently on the health benefits.

Many theories have been proposed to explain the association between greenspace exposure and health gains. The first hypothesis is that greenspace exposure may represent an opportunity for PA. PA is widely recognized as one of the most important protective factors of many NCDs [110], including cardiovascular diseases [111], hypertension [112], diabetes [113], obesity [114], mental disorders [32], and cancers [115,116]. However, according to some studies, higher health gains could be reached with outdoors, rather than indoors, PA. Outdoor PA allows for enhanced sunlight exposure, thereby facilitating vitamin D synthesis. Vitamin D is a lipid-soluble molecule acting as a hormone [16]. Among its many biological functions, vitamin D helps regulate calcium metabolism and exerts an immune-modulating and anti-inflammatory effect. Vitamin D deficiency has been associated with a wide range of immune-mediated diseases, such as diabetes, ischemic heart disease, Alzheimer’s, asthma and multiple sclerosis. Another hypothesis postulates that greenspace attendance increases social interactions and improves subjective well-being [117]. The fourth is the renowned “old friends hypothesis” [118]. The higher prevalence rates of allergies and immune-mediated disorders might be traced back to reduced stimuli by antigens and microbes, caused by reduced contact with the biodiversity-rich natural environments. This would imply that, on the contrary, increased exposure to natural habitats, and consequently to microbial biodiversity, determines a protective effect against infections and immune disorders.

Greenspaces can also influence social capital by providing a meeting place for users to develop and maintain neighbourhood social bonds [23,119]. Social interactions improve communication skills [120,121], thereby strengthening neighbourhoods’ social bonds, which dramatically affects perceived safety [120]. Policymaking efforts should be directed at tackling inequities in urban greenspaces access [122]. In addition to decreasing inequalities in terms of accessibility to green areas, it is necessary to incentivize the increase and improvement of characteristics such as the capillarity (through urban regeneration and greening of the available flat roofs) and the continuity of the green infrastructures, as well as the promotion of public–private collaboration in the maintenance of green areas in order to better involve the population and citizenship, with positive indirect mental health outcomes. Previous studies have shown how the main predictors of urban greenspaces use are quality and maintenance [44,72,75,102,103]. Low-income neighbourhoods are often underprivileged in terms of natural resources; even though urban greenspace might be present, they are often deteriorated and poorly maintained, with vandalized or dangerous areas [82]. In the early 2000s, scholars coined the term “environmental justice” [123] to illustrate spatial models where socioeconomic and environmental deprivation coexisted. Further research has shown how a lack of contact with restorative natural resources (such as urban greenspaces) is a social determinant of health inequities, especially in vulnerable, economically disadvantages subgroups [124]. Alongside the need for basic access to healthcare services, access to green environments is crucial for social justice. In this perspective, public greenspaces should be considered essential public health resources [101,106,107].

Our review underscores that mere urban greenspace presence is not enough to secure the desired health outcomes. On the contrary, important elements that need to be considered and reinvigorated are maintenance, access, and perceived security aspects. A pervasive determinant of both MH and PA-related health gains was the degree to which concrete, interactive activities were planned and disseminated to the general population. From this perspective, our results are significant for public health experts and policymakers involved in urban planning, community health promotion, and improvement of health and social equity [125]. Lastly, our results are consistent with previous and recent reviews [101,102,103], despite the fact that the review methodology and inclusion/exclusion criteria were different. For instance, a scoping review approach was used, in contrast with our systematic search. Moreover, we only included scientific literature, whereas another study also included grey literature [103]. Another difference is the geographical filter adopted. Indeed, in our study, we included general population living in the OECD area; on the contrary, Callaghan et al. [103] conducted a European-based review, while Lee et al. [102] and Wendelboe-Nelson et al. [101] did not apply geographical restrictions. Moreover, previous reviews generically referred to green space exposure, without focusing on urban green space, as in the current systematic review. Another different criterion used was the time filter. In particular, we restricted our search to articles published after 2000, whilst Callaghan [103] included studies until 2019. Whereas, since Wendelboe et al. [101] published their study in 2011, they could not include the last decade, and Lee et al. [102] which considers studies from 1990. Moreover, all the previous researches only focused on mental health/well-being; on the contrary, we included both physical activity and mental health (using several potential outcomes, such as, for instance, well-being, anxiety, stress, and etc.). Lastly, even if previous reviews searched in many electronic databases, the final number of included studies did not dramatically change, and more importantly, no differences in data interpretation have been detected.

### Strengths and Limitations

However, some limitations to our results generalization and external validity need to be acknowledged. Firstly, this was a systematic review, which was limited to only two databases. Nevertheless, the assessment of two databases is in line with the minimum requirements set by the PRISMA guidelines for systematic reviews. Secondly, we limited our search to articles published in English. However, since only one article was removed because of this language limitation, that in any case was not relevant to our topic, we are confident that our results are not affected by selection bias. Thirdly, in most of the cases, the authors used a cross-sectional or a before-after design, limiting the interpretability of the results. Moreover, the use of a cross-sectional design did not exclude reverse causality. Fourthly, the methodological quality of the included studies was below the cut-off for high quality. It was particularly true for interventional studies. Lastly, high heterogeneity was detected in both study design, outcome identification and outcome measures. MH outcomes were often grouped into macro-domains, such as depressive symptoms, anxiety levels, psychosocial stress, and even elusive categories, such as “perceived well-being”. The same degree of heterogeneity permeated the chosen psychometric scales. As for PA, although the results were often operationalized as METS (metabolic equivalents), there was heterogeneity in the tools used to derive such measures (accelerometers, SOPARC and others). However, our study also has important strengths. It is a systematic review that assessed more than 300 papers retrieved in two databases. Furthermore, our search was not restricted to only one outcome. Indeed, we reviewed articles establishing associations between several mental and physical health domains. Lastly, despite the weaknesses of the included studies, the results were coherent in retrieving the beneficial effects of urban green spaces and health (both physical activity and mental health).

## 5. Conclusions

Despite the above-mentioned limitations inherent to the current systematic review, we can state that the different studies identified have shown an almost univocal potential beneficial effect of urban greenspaces. Such an impact is to be ascribed, at least partially, to a complex relationship mediated by different personal and environmental factors. Nevertheless, such results need to be tailored to specific contexts, population characteristics, and the level of maintenance, accessibility and perceived security of individual urban greenspaces. Future research should help reduce the high methodological heterogeneity, and the use of validated tools should be encouraged. Importantly, urban greenspaces exposure should be measured more accurately by future research. According to what is suggested and encouraged by the World Health Organization (WHO) regarding the “urban green spaces and health” issues, both green areas and the exposure to it should be deeply analyzed, through specific indicators. Those indicators, for instance, could be related to: (i) indicators of green space availability (i.e., density and diversity of trees or percentage of green space by area, using also GIS-based data); (ii) indicators of green space accessibility (proximity to an urban park or proportion of green space from residence, using also GIS-based data); (iii) indicators of green space usage (community-based survey about both frequency of attendance, and time and methods of the green areas’ use and accessibility, different for types of users, or using global positioning system technology, or digital gate count).

Indeed, almost all the included studies took indirect indexes, such as residential closeness, as a proxy indicator of urban greenspaces exposure. All these elements can improve comparability and reduce uncertainty. In this respect, joining research efforts into consortia or multicentric studies is a plausible solution.

## Figures and Tables

**Table 1:** Descriptive characteristics of the included studies stratified by health outcome (PA and mental health) and listed in alphabetical order and based on study design.

Physical Activity											
Author, Year [Ref]	Study Period	Country	Study Design	Type of Greenspace	City	Sample Size	Statistical Analysis	Tool Used to Measure PA	Outcome Domain	Main Results	QS/9
Observational Studies											
Cerin E., 2017 [70]	2002–2011	BE, BR, CO, CZ, DK, HK, MX, NZ, UK, US	Cross-sectional	PUM	Ghent, Curitiba, Bogotá, Olomouc, Aarhus, Hong Kong, Cuernavaca, North Shore, Waitakere, Wellington, Christchurch, Stoke-On-Trent, Seattle, Baltimore	6712	Mixed-model regression measures	Accelerometer	PA regardless of the setting	MVPA in urban parks was lower in the late evening/night (1.2 ± 4.0 min/h) and higher in the afternoon (3.0 ± 4.0 min/h) of weekend days	9
Cohen D.A., 2014 [72]	2006–2008	US	Quasi-experimental post-only assessment	PUM	Los Angeles	n.a.	CEA	SOPARC	PA in greenspace only	Average visitor number: higher for pocket parks (n = 147) than larger UGS (n = 134). Total PA performed shows opposite trend: 324 vs 374 METs)	8
Cohen D.A., 2017 [74]	2014	US	Cross-sectional	PUM	25 US cities > 100,000 residents each	n.a.	LRM	SOPARC	PA in greenspace only	Parks with walking loops attract 80% (95% CI: 42–139%) [*p* < 0.001] more visitors per hour and show increased levels of MVPA with 90% more MET-hours (95% CI: 49–145%) [*p* < 0.001] than unequipped counterparts	8
Copeland J.L., 2017 [75]	2015	CA	Cross-sectional	PUM	Lethbridge	1646	T-test	SOPARC	PA in greenspace only	Only 2.7% of adult visitors used fitness equipments for PA	5
Parra D.C., 2019 [83]	2018	US	Cross-sectional	RUGF	Wellston	599	Chi^2^	SOPARC	PA in greenspace only	Children and middle-aged adults represented 41.1% and 50.3% of total park users, respectively. A total of 47% of them practised MVPA, 22% LPA and 30% was sedentary	5
Ramírez P.C., 2017 [86]	2015	CO	Cross-sectional	RUGF	Bucaramanga	6722	Chi^2^	SOPARC	PA in greenspace only	Women more prone to use outdoor gyms than men (51.7% against 48.3%) and to practise intense PA levels (W = 53.5%; M = 46.5%)	4
Roemmich J.N., 2018 [88]	2014	US	Cross-sectional	PUM, UFAP	Grand Forks, ND and East Grand Forks, MN	5486	T-test	SOPARC	PA regardless of the setting	Rural parks dwellers display lower MPA prevalence than urban parks (34%, n = 240 against 48%, n = 1828)	9
Sallis J.F., 2016 [90]	2002–2011	BE, BR, CO, CZ, DK, HK, MX, NZ, UK, US	Cross-sectional	RUGF	Ghent, Curitiba, Bogotá, Olomouc, Aarhus, Hong Kong, Cuernavaca, North Shore, Waitakere, Wellington, Christchurch, Stoke-On-Trent, Seattle, Baltimore	10,008	SEV MEV GAMMs	Accelerometer	PA regardless of the setting	Positive correlation between PA and urban parks presence within 0.5 Km of the participants’ home in Ghent (exp[β] = 1.772; 95% CI: 1.177–2.669; *p* = 0.006) and Seattle (exp[β] = 2.064; 95% CI: 1.399–3.045; *p* < 0.001)	8
Spengler J.O., 2011 [93]	2005	US	Cross-sectional	PUM, SUG, RUGF	Tampa, Chicago	3410	Multilevel regression	SOPLAY	PA in greenspace only	Children perform MVPA most frequently (56.2% boys, 55.7% girls, *p*-value n.a.) in parks with playgrounds than in all other UGS	6
Suau L.J., 2012 [94]	2005	US	Cross-sectional	PUM, SUG, RUGF	Tampa, Chicago	9454	Multilevel regression	SOPLAY	PA in greenspace only	In Chicago’s parks, PA was greater in African American (F = 5.027; *p* < 0.01) and high-income neighborhoods (F = 5.027; *p* = 0.002)	4
Author, year [Ref]	Study period	Country	Study design	Type of greenspace	City	Sample size	Statistical analysis	Tool used to measure PA	Outcome domain	Main results	QS/21
Park S., 2018 [82]	2013–2015	US	Ecological	PUM	Los Angeles	52,596 MPA, 5975 VPA	Chi^2^	Accelerometer	PA in greenspace only	The proportion of park use time spent in MVPA (33.1%) was lower than the city-level average (35%)	15/21
Interventions											
Author, year [Ref]	Study period	Country	Study design	Type of greenspace	City	Sample size	Statistical analysis	Tool used to measure PA	Outcome domain	Main results	QS
Andersen H.B., 2017 [64]	2010; 2012 pre and post intervention	DK	Pre-post intervention	PUM, SUG	Copenhagen	673	Wilcoxon’s rank-sum test	Accelerometer, GPS, GIS	PA regardless of the setting	After intervention, 4.5 min/day increase in adolescents’ greenspace PA (95% CI: 1.8, 7.2; *p* < 0.001)	Fair
Cohen D.A., 2013 [71]	2010–2011	US	Randomized controlled trial	PUM	Albuquerfque, Chapel Hill, Columbus, Philadelphia	36,000	LRM	SOPARC	PA in greenspace only	Programmed activities (IRR: 1.79; *p* < 0.001) and the number of activity facilities (IRR: 1.13; *p* = 0.01) are associated with higher park use. Programmed activities (β = 192 ± 37; *p* < 0.001) and number of activity facilities (β = 28 ± 27; *p* = 0.30) are associated also with higher energy expended in the park too	Some
Cohen D.A., 2017 [73]	2013–2015	US	Randomized cluster trial	PUM	Los Angeles	52,310	DID models	SOPARC	PA in greenspace only	Free classes arm attracted more than twice park visits than the frequent user program. (*p*-value n.a.). (Among free classes arm it was show a 10% increase in total number of park users, more than twice the increasing percentage in frequent user program arm total number (*p*-value n.a.)	
Tester J., 2009 [95]	2006–2007	US	Pre-post intervention	SUG	San Francisco	2041	T-test	SOPARC	PA in greenspace only	Significant increase in visitors for PA among children (*p* < 0.05) and adults of both genders (*p* < 0.001) following parks’ renovations	
Mental Health											
Author, year [Ref]	Study period	Country	Study design	Type of greenspace	City	Sample size	Statistical analysis	Tool used to measure MH	Outcome domain	Main results	QS/9
Observational Studies											
Andrusaityte S., et al., 2020 [65]	2007–2009	LT	Cross-sectional	PUM	Kaunas	1489	multivariate logistic regression	SDQ	Well-being/quality of life	Each increasing hour/week of park visits shows a non-significant association with mental difficulties: (aOR = 0.98 (0.96–1.01, [*p* < 0.05])	4
Astell-Burt T., et al., 2019 [66]	2006–2015	AU	Cohort	Total greenspace	Sydney, Wollongong, and Newcastle	4786	multilevel logistic regression	K10	Psychosocial stress	A 30% increase in total greenspace percentage is protective against both prevalent K10 psychological distress (aOR = 0.69 (0.47–1.02) [*p* = 0.03]) and incident K10 psychological distress (aOR = 0.46 (0.29–0.69) [*p* < 0.001])	8
Balseviciene B., et al., 2014 [67]	2007–2009	LT	Cross-sectional	PUM	Kaunas	1468	LRM	SDQ	Well-being/quality of life	Proximity to city parks associated with increased mental difficulties in the lower maternal education subgroup (beta coefficient = 1.293, *p* < 0.05, R = 0.444).	8
Bixby H., et al., 2015 [68]	2002–2009	UK	Cross-sectional	PUM, SUG, RUGF, UFAP and BS	50 largest cities in England	5222	Poisson linear regression	Mortality data: ICD-10 codes X60–84	Suicide	Comparing quintiles 1 vs. 5 of greenspace coverage. RR of death from suicide was 1.02 (0.86–1.23) in men and 1.10 (0.77–1.57) in women [*p* < 0.05 for both].	5
Camargo D.M., et al., 2017 [69]	2015	CO	Cross-sectional	PUM and SUG	Bucaramanga	1392	Multiple regression	EQ5D-5L	Well-being/quality of life	Positive associations between quality of life and: tree conditions status -> aPR = 1.20 (1.07–1.34), perceived safety -> aPR = 1.22 (1.04–1.44) [*p* < 0.05 for both]	8
Feda D.M., et al., 2015 [77]	2008–2010	US	Cross-sectional	PUM, SUG and RUGF	New York and Buffalo	68	Multiple regression analysis	PSS	Psychosocial stress	Percentage of park area predicted perceived stress β = −62.573, [*p* < 0.03]	8
Guite H.F., et al., 2006 [78]	n.a.	UK	Cross-sectional	Not specified	Greenwich (London)	2696	mutivariate logistic regression	SF-36v2	Well-being/quality of life	Dissatisfaction with open UGS access significantly associated with lowest quartile for well being and quality of lifeOR = 1.69 (1.05–2.74)	8
Ihlebæk C., et al., 2018 [79]	2000–2001	NO	Cross-sectional	PUM, RUGF, UFAP, BS	Oslo	8638	Logistic regression	Not validated questionnaire	General mental health	With enhanced exposure to UGS, significant drop in MH disorders prevalence in women (−6% *p* = 0.049) but not in men (−2.5% *p* = 0.129)	6
Lee H.J., et al., 2019 [80]	2015	KR	Cross-sectional	PUM, SUG, UFAP and BS	7 metropolitan areas in Korea	11,408	Binary logistic regression analysis	Not validated questionnaire	Depression and Psychosocial stress	Inverse relationship between stress levels, depressive symptoms and urban green area ratio (*p* < 0.005)	7
Pope, D., et al., 2018 [84]	2009–2013	UK	Cross-sectional	PUM	Sandwell	1680	Multivariable logistic regression	GHQ-12	Psychological stress	Wider greenspace accessibility associated with reduced PD [OR = 0.13 (0.42, 0.94)]	6
Reklaitiene, R., et al., 2014 [87]	2006–2008	LT	Cross-sectional	PUM	Kaunas	6944	Multiple logistic regression	CES-D10	Depressive symptoms	Living >300 m away from UGS and using them ≥4 h/week showed higher odds 1.92 (1.11–3.3) and 1.68 (0.81–3.48) of depressive symptoms	6
Ruijsbroek, A., et al., 2017 [89]	2013	ES, NL, LT, UK	Cross-sectional	NGS	Barcelona, Doetinchem, Kaunas, Stoke-on-Trent	3771	Multilevel regression analyses	MHI-5	Nervous and feelings of depression in the past month	Only in Barcelona, NGS quantity was associated with better MH status (1.437 ± 0.71) *p* < 0.05	9
Van Dillen, S.M., et al., 2012 [96]	2007	NL	Cross-sectional	SUG	80 Dutch urban neighborhoods	1641	Multilevel regression	MHI-5	General mental health	Perceived general health and green areas, had a significant interaction with the following parameters: quantity = 0.27 (0.013), quality = 0.126 (0.066), interaction term = 0.084 (0.040)	5
Zhang, Y., et al., 2015 [97]	2014	NL	Cross-sectional	PUM; SUG	Groningen	223	Multivarite ANOVA	MHI-5	General mental health	Differences in neighborhood have a positive and significant influenceon mental health, β = 0.15, t(245) = 2.10, *p* < 0.05	5
Author, year [Ref]	Study period	Country	Study design	Type of greenspace	City	Sample size	Statistical analysis	Tool used to measure MH	Outcome domain	Main results	QS/21
Nutsford, D., et al., 2013 [81]	2008–2009	NZ	Ecological	PUM, SUG, RUGF, UFAP	Auckland City	319,521, of which 7552 cases	Negative binomial regression models	Record linkage (treatment)	Mood state and general anxiety	Better access UGS access, and decreased distance (less than 3km) reduced the risk of anxiety/mood disorders treatment by 4% and 3% respectively (*p* < 0.01)	12/21
Interventions											
Author, year [Ref]	Study period	Country	Study design	Type of greenspace	City	Sample size	Statistical analysis	Tool used to measure MH	Outcome domain	Main results	QS
Coventry P.A., et al., 2019 [76]	2017	UK	Pre-post intervention	PUM	York	45	One-way ANOVA + Bonferroni correction for multiple comparisons	SWEMWBS, UWIST-MACL	Affective/general and well-being/quality of life/ stress and (physical) arousal	UWIST-MACL mean difference (pre-post intervention stress levels across all participants at all locations) of −3.53 (4.79–2.28) [*p* < 0.001]	Fair
Pratiwi, P.I., et al., 2019 [85]	2019	JP	Pre-post intervention	PUM	Matsudo	24	Wilcoxon’s rank-sum test	POMS-STAI	Mood state and general anxiety	POMS scores: 0.71 in spring and 0.896 in summer. STAI score 0.896 and 0.933 respectively	Fair
Song, C., et al., 2015 [91]	2014	JP	Pre-post intervention	SUG	Kashiwa City	20	Wilcoxon’s rank-sum test	STAI	Anxiety and mood state	STAI score was 19.3% significantly lower after the urban park walk than after the city area walk (urban park: 39.0 ± 6.3; city area: 48.4 ± 7.5; *p* < 0.01)	Fair
South, E.C., et al., 2018 [92]	2011–2014	US	Randomized cluster trial	PUM	Philadelphia	149	Pairwise comparison using time serious regression	K6	General mental health and depression	ITT analysis of the greening intervention demonstrated a non-significant reduction in overall self-reported poor MH with respect to non-intervention (−62.8%; 95% CI, −86.2% to 0.4%; *p* = 0.051) but a significant reduction in depressive symptoms (−41.5%; 95%CI, −63.6% to −5.9%; *p* = 0.03)	Low

AU: Australia; BE: Belgium; BR: Brazil; BS: “Blue” spaces; CA: Canada; CEA: Cost-effectiveness analysis; CES-D10: Center for the Epidemiological Studies of Depression Short Form 10-items; CI: Confidence Interval; CO: Colombia; CZ: Czech Republic; DID: Difference-in-differences; DK: Denmark; EQ5D-5L: EuroQol 5 Dimensions-5 Levels; ES: Spain; F: Fisher’s F-test distribution; GAMM: Generalized Additive Mixed Models; GHQ-12: General Health Questionnaire-12; GIS: Geographic Information Systems; GPS: Global Positioning Systems; Exp: Expected; HK: Hong Kong; ICD-10: International Statistical Classification of Diseases and Related Health Problems 10; IRR: Incidence Rate Ratio; ITT: intention-to-treat JP: Japan; K6: Kessler-6-Psychological Distress Scale; K10: Kessler Psychological Distress Scale; KR: Korea; LRM: Linear regression model; LT: Lithuania; M: Men; METS: Metabolic Equivalents; MEV: Multiple Environmental Variable; MH: mental health; MHI-5: The Revised Mental Health Inventory-5; MN: Minnesota; MPA: Moderate-intensity Physical Activity; MVPA: Moderate/Vigorous Physical Activity; MX: Mexico; N: Number; ND: North Dakota; NL: Netherlands; NZ: New Zealand; OR: Odds Ratio; PA: Physical Activity; POMS-STAI: Profile of Mood States—State Trait Anxiety Inventory; PSS: Perceived Stress Scale; PUM: Parks and urban meadows; QS: Quality Score; RR: Relative Risk; RUGF: Recreational and urban gardening facilities; SDQ: Strengths and Difficulties Questionnaire; SEV: Single Environmental variable; SF-36v2: SF36 subscales for mental health; SOPARC: System of Observing Play and Recreation in Communities; SOPLAY: System for Observing Play and Leisure Activity in Youth; STAI: State-Trait Anxiety Inventory; SUG: “small” urban greenspaces; SWEMWBS: Short Warwick–Edinburgh Mental-Wellbeing Scale; UFAP: Urban forests and agricultural parks; UGS: urban greenspace; UK: United Kingdom; US: United States; UWIST-MACL: Measured by the University of Wales Institute of Science and Technology -Mood Adjective Checklist; VPA: Vigorous Physical Activity; W: Women; aPR: adjusted Prevalence Ratio; aOR: adjusted Odds Ratio; n.a.: not available; β: β coefficient.

## Data Availability

All data are presented in the current manuscript (text, tables, and supplementary material).

## Supplementary Materials

The following are available online at https://www.mdpi.com/article/10.3390/ijerph18105137/s1, Table S1: Detailed search strategy, Table S2: Definitions of urban greenspaces used, Table S3: Detailed description of inclusion/exclusion criteria according to a Population, Exposure, Outcomes and Study design (PEOS), Table S4: Articles assessed in full and excluded with reasons, Table S5: Quality assessment of the included studies, in alphabetical order and stratified by study design.
