# Supplementary material for: Association between Urban Greenspace and Health: A Systematic Review of Literature
Source: Int J Environ Res Public Health. 2021 May 12;18(10):5137. doi: 10.3390/ijerph18105137 (PMC8150317; doi:10.3390/ijerph18105137)
Supplement: Supplementary file 1 [file ijerph-18-05137-s001.zip › Supplementary Tables.pdf]

Supplementary Table S1. Detailed search strategy.

**PubMed/MEDLINE**	((("urban green*"[Tiab] OR "parks"[Tiab] OR "parks, recreational"[MeSH Terms]) AND ("cities"[MeSH Terms] OR "urban health"[MeSH Terms] OR "mental unhealthy days"[Tiab]) AND ("environment design"[MeSH Major Topic]))) NOT ("review"[Publication Type] OR "meta analysis"[Publication Type] OR "journal article"[Publication Type]) OR "letter"[Publication Type]) OR "editorial"[Publication Type]) OR "comment"[Publication Type]) OR "preprint"[Publication Type]))
**EMBASE**	Health[Tiab] AND (city[Tiab] OR urban Green[Tiab] OR park[Tiab] OR garden*[Tiab] OR 'urban green space'[Tiab] OR parks[Tiab] OR 'recreational park') AND ('city' [Emtree] OR 'urban health'[Emtree])
**Time filter**	From 2000/01/01 to 2020/09/30
**Language limit**	"english"[Language]

Supplementary Table S2. Comprehensive definitions of urban greenspaces used.

**Parks and urban meadows (PUM)**	Green areas of a mean extent (Area: > 10 hectares that is 100.000 m2) located in urban areas.<br>Urban parks can include different compartments with different functions such as gardens, playgrounds, sports fields, cultural and recreational infrastructures and amenities, paths, public restrooms, etc.<br>Influence area: the city (accessibility by walking, bike, car, public transport).
**"small" urban greenspaces (SUG)**	Green areas (gardens, pocket parks, playgrounds) scattered in an urban settlement with an extent of about 50.000 m2 or smaller. They include public gardens pocket parks, playgrounds used by the citizens of a neighbourhood or district for recreation.<br>These green spaces have a relatively high attendance, despite their limited extent. They can host trees, shrubs, hedges, meadows, flowerbeds,

	playgrounds, walkways, benches, small picnic areas, community gardens, etc... Influence area: distance between 500 to 1500 m; walking accessibility
**Neighborhood green spaces (NGS)**	– small areas - pocket parks, playgrounds, gardens, squares - used by the close neighbourhood (particularly child, elderly) for recreation and relax. The extent of these areas is about 5.000 m2 or less<br>Influence area: distance between 100 to 200 m; walking accessibility.
**Urban forests and agricultural parks (UFAP)**	Urban forest are woods that grow around or close to an urban area or in suburban areas.<br>Agricultural parks: agricultural land of cultural, historical, environmental interest located close to a city or in the sub-urban area. They can also represent a traditional historical landscape.<br>These areas have a large extent and can include areas protected because of their environmental, cultural or landscape value<br>Influence area: the whole municipality territory and the suburbs (accessibility by bike, by public transport, by car).
**Recreational and urban gardening facilities (RUGF)**	Green areas with very specific function, such as open space sports facilities, school grounds, educational and thematic parks, green spaces in healthcare settings, community gardens (green areas managed by the citizens of a neighbourhood for fruit or vegetable production and/or for aesthetic purpose).
**"Blue" spaces (BS)**	Urban areas characterized by water bodies and watercourses including river, lake and coastal sides, available to citizens for recreational use.

Supplementary Table S3. Detailed description of inclusion/exclusion criteria according to a Population, Exposure, Outcomes and Study design (PEOS).

Search strategy	Details
Inclusion criteria	P: general population (both sex and any age) living in the OECD area<br>I/E: exposure to publicly accessible urban greenspace<br>C: no exposure or less exposure to urban greenspace<br>O: physical activity (objectively measured) and mental health<br>S: Trials, cohort, case-control, cross-sectional, ecological, quasi-experimental

Exclusion criteria	P: general population out of OECD area<br>I/E: exposure to not urban greenspace/exposure to urban greenspace not publicly accessible<br>O: other health outcomes, self-reported physical activity (i.e. not objectively measured)<br>S: non-original papers (opinion paper, review article, commentary, letter, protocols, article without quantitative data)
Language filter	English
Time filter	From 2000
Databases	PubMed/Medline; EMBASE

OECD: Organization for Economic Co-operation and Development

Supplementary Table S4 Articles assessed in full and excluded with reasons

References	n. studies	Reasons
Astell-Burt, T., et al. 2014(44)	1	No publicly-available greenspace (residential greenspace)
Akpinar, A., et al. 2017(45); An, S., et al. 2013(46); Bezold, C. P., et al. 2017(47); Burrows, E., et al. 2018(48); de Vries, S., et al. 2013(49); Derose, K. P., et al. 2014(50); Derose, K. P., et al. 2018 (51); Esliger, D. W., et al. 2012(52); Hamilton, K., et al. 2017(53); Hillsdon, M., et al. 2006(54); McCormack, G. R., et al. 2016(55); Schroeder, K., et al. 2019(56); Tamosiunas, A. et al. 2014(57); Völker, S., et al. 2015(58); Winter, P. L., et al. 2019(59)	15	Non-objectively measured physical activity
Cohen, D. A., et al. 2013 (60), Cohen, D. A., et al. 2019 (61); Cutts, B. B., et al. 2009 (62); Greenberg, M. R., et al. 2005 (63)	4	Assess walkability/accessibility/usability

Supplementary Table S5 Quality assessment of the included studies, in alphabetical order and stratified by study design.

**Cross-sectional/ Cohort studies, quality assessed by NOS**										
**Author year [Ref]**	**Selection**				**Comparability**		**Outcome/Exposure^**			**Tot/9**
	**Item 1**	**Item 2**	**Item 3**	**Item 4**	**Item 5a**	**Item 5b**	**Item 6**	**Item 7**	**Item 8**	
**Andrusaityte, 2020[65]**	-	-	-	*	*	*	-	*	n.a.	4
**Astell-Burt, 2019[66]**	*	*	*	*	*	*	*	*	-	8
**Balseviciene, 2014**	*	*	-	*	*	*	**	*	n.a.	8
**Bixby, 2015[68]**	-	*	-	*	*	-	*	*	n.a.	5
**Camargo, 2017[69]**	*	*	-	*	*	*	**	*	n.a.	8
**Cerin, 2017[70]**	*	*	-	*	*	*	**	*	n.a.	9
**Cohen, 2014[72]**	*	*	-	*	*	*	**	*	n.a.	8

**Cohen, 2017[74]**	*	*	-	*	*	*	**	*	n.a.	8
**Copeland, 2017[75]**	*	-	-	*	-	-	**	*	n.a.	5
**Feda, 2015[77]**	*	*	-	*	*	*	**	*	n.a.	8
**Guite, 2006[78]**	*	*	-	*	*	*	**	*	n.a.	8
**Ihlebæk C., 2018[79]**	*	*	-	*	*	*	-	*	n.a.	6
**Lee, 2019[80]**	*	*	*	*	*	*	-	*	n.a.	7
**Parra, 2019[83]**	-	-	*	*	*	*	-	*	n.a.	5
**Pope, 2018[84]**	*	*	-	*	*	*	-	*	n.a.	6
**Ramirez, 2017[86]**	-	-	-	*	*	*	-	*	n.a.	4
**Reklaitiene, 2014[87]**	*	*	-	*	*	*	-	*	n.a.	6
**Roemmich, 2018[88]**	*	*	*	*	*	*	**	*	n.a.	9
**Ruijsbroek, 2017[89]**	*	*	*	*	*	*	**	*	n.a.	9
**Sallis, 2016[90]**	*	*	*	*	*	-	**	*	n.a.	8
**Spengler, 2011[93]**	-	*	-	*	*	-	**	*	n.a.	6
**Suau, 2012[94]**	-	-	-	*	-	-	**	*	n.a.	4
**Van Dillen, 2012[96]**	-	*	-	*	*	*	-	*	n.a.	5
**Zhang, 2015[97]**	*	*	-	*	-	*	-	*	n.a.	5

Ecological studies, quality assessed with the checklist proposed by Dufault B et al. 2011

	**Study design**								**Statistical methodology**				**Quality of reporting**			**Tot/21**
**Park, 2018[82]**	2	1	0	1	1	1	2	1	1	1	1	1	1	1	0	15
**Nutsford, 2013[81]**	1	1	1	1	0	0	1	1	1	1	1	0	1	1	1	12

Experimental studies, quality assessed with the RoB2 suggested by the Cochrane Collaboration 2019

	**Domain 1**	**Domani 2a**	**Domain 2b**	**Domain 3**	**Domain 4**	**Domain 5**	**Tot**
**Cohen, 2013[71]**	Some	Some	n.a.	Low	Some	Some	Some
**Cohen, 2017[73]**	Some	Some	n.a.	Low	Some	Some	Some
**South, 2018[92]**	Low	Low	n.a.	Low	Low	Low	Low

Experimental studies, pre-post intervention quality assessed with the NIH tool

	**Item1**	**Item2**	**Item3**	**Item4**	**Item5**	**Item6**	**Item7**	**Item8**	**Item9**	**Item10**	**Item11**	**Item12**	**Tot**
**Andersen, 2017[64]**	yes	yes	yes	n.r.	yes	yes	yes	n.r.	yes	yes	no	n.a.	Fair
**Coventry, 2019[76]**	yes	n.r.	no	n.r.	no	yes	yes	n.r.	yes	yes	no	n.a.	Fair
**Pratiwi, 2019[85]**	yes	yes	no	n.r.	no	yes	yes	n.r.	yes	yes	no	n.a.	Fair
**Tester, 2009[95]**	yes	yes	yes	n.r.	yes	yes	yes	n.r.	yes	yes	no	n.a.	Fair

**Song, 2015[91]**	yes	n.r.	no	n.r.	no	yes	yes	n.r.	yes	yes	no	n.a.	Fair

^based on study design. For case-control the NOS assessed the exposure, for cross-sectional and cohort studies the outcome.
n.a. not applicable, n.r.: not reported

Dufault B, Klar N. The quality of modern cross-sectional ecologic studies: a biblio-metric review. Am J Epidemiol. 2011;174(10):1101-7.

Sterne JAC, Savović J, Page MJ, Elbers RG, Blencowe NS, Boutron I, Cates CJ, Cheng H-Y, Corbett MS, Eldridge SM, Hernán MA, Hopewell S, Hróbjartsson A, Junqueira DR, Jüni P, Kirkham JJ, Lasserson T, Li T, McAleenan A, Reeves BC, Shepperd S, Shrier I, Stewart LA, Tilling K, White IR, Whiting PF, Higgins JPT. RoB 2: a revised tool for assessing risk of bias in randomized trials. BMJ 2019; 366: l4898.

The National Institutes of Health (NIH). Quality Assessment Tool for Before-After (Pre-Post) Studies With No Control Group 2020 [Available from: https://www.nhlbi.nih.gov/health-topics/study-quality-assessment-tools.
